# Prenatal Metal Exposures and Infants’ Developmental Outcomes in a Navajo Population

**DOI:** 10.3390/ijerph19010425

**Published:** 2021-12-31

**Authors:** Sara S. Nozadi, Li Li, Li Luo, Debra MacKenzie, Esther Erdei, Ruofei Du, Carolyn W. Roman, Joseph Hoover, Elena O’Donald, Courtney Burnette, Johnnye Lewis

**Affiliations:** 1 Health Sciences Center, College of Pharmacy, University of New Mexico, Albuquerque, NM 87131, USA; dmackenzie@salud.unm.edu (D.M.); EErdei@salud.unm.edu (E.E.); cwroman@salud.unm.edu (C.W.R.); EODonald@salud.unm.edu (E.O.); jlewis@cybermesa.com (J.L.); 2 Department of Mathematics and Statistics, University of New Mexico, Albuquerque, NM 87131, USA; llis@unm.edu; 3 Department of Internal Medicine, UNM Comprehensive Cancer Center, University of New Mexico, Albuquerque, NM 87131, USA; LLuo@salud.unm.edu; 4 Department of Biostatistics, University of Arkansas for Medical Sciences, Little Rock, AR 72205, USA; RDu@uams.edu; 5 Social Science and Cultural Studies, Montana State University Billing, Billings, MT 59101, USA; joseph.hoover@msubillings.edu; 6 Munroe-Meyer Institute, University of Nebraska Medical Services, Omaha, NE 68106, USA; courtneyburnette@gmail.com

**Keywords:** developmental outcomes, metal exposure, environmental exposure, Navajo Nation

## Abstract

Early-life exposure to environmental toxicants can have detrimental effects on children’s neurodevelopment. In the current study, we employed a causal modeling framework to examine the direct effect of specific maternal prenatal exposures on infants’ neurodevelopment in the context of co-occurring metals. Maternal metal exposure and select micronutrients’ concentrations were assessed using samples collected at the time of delivery from mothers living across Navajo Nation with community exposure to metal mixtures originating from abandoned uranium mines. Infants’ development across five domains was measured at ages 10 to 13 months using the Ages and Stages Questionnaire Inventory (ASQ:I), an early developmental screener. After adjusting for effects of other confounding metals and demographic variables, prenatal exposure to lead, arsenic, antimony, barium, copper, and molybdenum predicted deficits in at least one of the ASQ:I domain scores. Strontium, tungsten, and thallium were positively associated with several aspects of infants’ development. Mothers with lower socioeconomic status (SES) had higher lead, cesium, and thallium exposures compared to mothers from high SES backgrounds. These mothers also had infants with lower scores across various developmental domains. The current study has many strengths including its focus on neurodevelopmental outcomes during infancy, an understudied developmental period, and the use of a novel analytical method to control for the effects of co-occurring metals while examining the effect of each metal on neurodevelopmental outcomes. Yet, future examination of how the effects of prenatal exposure on neurodevelopmental outcomes unfold over time while considering all potential interactions among metals and micronutrients is warranted.

## 1. Introduction

The Navajo Nation, comprising lands in Arizona, New Mexico, and Utah, was heavily mined for uranium in order to supply World War II and Cold War nuclear weapons’ research and programs [1,2]. Extraction of over 30 million tons of uranium ore through four decades left a legacy of ~500 abandoned uranium mines across the Western US and over 1000 associated waste features across the Navajo Nation alone, resulting in decades of exposures of Navajo Nation residents to uranium and a wide range of co-occurring metals, including arsenic, cadmium, copper, and lead [3].

Exposure to uranium as well as co-occurring metals from these abandoned mines and mine waste features has been linked with risks for a range of adverse health outcomes [1,4,5,6,7]. The Navajo Birth Cohort Study (NBCS) was initiated in 2010 to respond to Navajo community members’ long-held concerns about the potential impacts on future generations exposed to uranium mines and mine wastes, with a specific focus on neurodevelopmental outcomes [4,8]. In the current study, we examined samples from the NBCS for associations between in utero maternal exposure (measured through biomonitoring of a panel of 35 metals and metalloids) and infants’ neurodevelopmental outcomes (assessed using developmental screening of infants between 10 and 13 months) in mother–infant pairs from mined and unmined regions that encompass a broad range of exposures. The developmental period of 10–13 months was chosen because elevated developmental delays around 10 months of age were observed in previous analysis of data from NBCS [9]. The subset of co-occurring metals assessed in the current study includes uranium, lead, arsenic, cadmium, manganese, copper, barium, cobalt, cesium, molybdenum, antimony, strontium, tin, thallium, and tungsten. Previous analysis of biomonitoring samples from NBCS has shown that most of these select metals were detected in women of childbearing age with the median and 95th percentile concentrations exceeding respective values from the CDC National Health and Nutrition Examination Survey (NHANES), with concentrations relatively stable throughout the first and third trimesters [10]. In addition to these metals, we also explored the effects of three micronutrients important for normal growth and development, including selenium, zinc, and iodine.

Evidence on the effects of individual metals to developmental outcomes in previous literature varies substantially across different metals common in NBCS pregnant mothers. For example, exposures to lead, arsenic, manganese, and cadmium are consistently found to have negative effects on children’s verbal and non-verbal cognition, behavior problems, and motor skills, even after adjusting for other environmental and individual risk factors [11,12,13,14,15,16,17,18,19,20]. Adverse developmental outcomes associated with metals such as lead have been observed at very low concentrations, even lower than those in recommendations previously set forth by agencies such as the U.S. Centers for Disease Control and Prevention (CDC). These low-dose relationships have led agencies to lower their reference level for safe level of exposure [21]. Evidence from animal and epidemiological studies has demonstrated that these metals may accumulate in and affect different parts of the brain through diverse mechanisms, including disruptions in pituitary and hippocampal signaling and alterations in synaptic function [22,23]. There is evidence indicating that effects of some of these metals can start in utero because they easily cross the placental barrier to adversely impact fetal development [10,24,25].

Limited evidence is available for other metals frequently detected in NBCS participants such as uranium, thallium, molybdenum, copper, and tungsten. Yet, the absence of data does not equate to absence of effects, especially given the high potential for exposure in at-risk communities [26,27]. Some of these metals have U-shaped dose-response curves whereby they are essential for optimal growth and development in small doses, e.g., molybdenum and copper, but can be harmful at high doses. For example, findings from a recent study have shown a slightly U-shaped association between copper concentrations in mothers and children’s attention deficit-hyperactivity diagnosis status [28].

In the current study, we also considered the effects of the micronutrients iodine, zinc, and selenium. These micronutrients are important for normal growth but deficient in our population [29]. Iodine deficiency has been recognized as detrimental and damaging for organogenesis and brain development in the fetus [30,31]. Recent investigations also have suggested zinc may play a protective role in exposures to other metals. Zinc deficiency in low-income preschool-aged children was associated with higher blood lead concentrations and lower cognitive ability when compared to children in the same population who were zinc-sufficient with similar lead exposure [32]. For selenium, multiple studies have observed stronger cognitive and motor abilities in children whose mothers had higher blood concentrations of selenium [33,34,35].

The impact of co-exposure to environmental toxicants on health outcomes within communities is an issue that has received considerable attention over the past decade [36]. The field of chemical mixture analysis has been dominated by use of regression models to identify association and interaction effects of multiple co-occurring exposures with limited consideration of the underlying complex causal relationships. However, including co-occurring metal exposures in regression models without examining the underlying causal framework could lead to amplified bias. There is increasing research interest in developing analytical approaches for metal mixture analysis to unravel complex causal mechanisms [37]. Clustering analysis of metal mixtures can provide valuable information on a mixture profile associated with adverse health outcomes within a specific community or situation and even allow for parsing of the metal interactions in mixtures of that specific composition. However, clustering analysis describes the clustered effects of multiple metals on health outcomes, which is not designed to estimate the causal effects of individual metal exposures [37]. Additionally, the metal mixture profile is likely to be population specific, which is then hard to generalize to other populations where specific metals and their proportions in a mixture would likely vary. Finally, a cluster of metals may include metals with antagonistic effects on health outcomes of interest, resulting in limited or conflicting information to inform risk reduction strategies.

In the current study, we aimed to overcome the limitations in existing single metal and cluster analyses through utilization of a statistical causal inference technique coupled with a priori causal frameworks to estimate the effects of an individual metal on health outcomes while controlling for other potential residual confounding effects due to co-exposed metals. Estimating the effects of an individual metal on health outcomes while taking into account the effects of other co-exposures is necessary for identifying environmentally relevant and causal associations [38,39]. Appropriately controlling for metal co-exposures will reduce bias in estimating exposure effects on health outcomes and, in addition, identify the direct association of each metal with outcome. The identification of such effects has the potential to inform strategies and policies to reduce metal exposure risk, important for at-risk populations.

## 2. Materials and Methods

### 2.1. Study Population

Participants whose data were used in the analysis were mothers and their infants enrolled in the NBCS. The NBCS examines the effects of pre- and postnatal metal exposures on birth outcomes and children’s neurodevelopmental outcomes [10,26,27]. Study inclusion criteria for mothers were (1) a confirmed pregnancy; (2) being between 14 and 45 years of age; (3) willingness to deliver at one of six participating Navajo Area Indian Health Service or PL-638 hospitals; and (4) residency on the Navajo Nation for at least 5 years. The study was approved by the Navajo Nation Human Research Review Board (NNHRRB) and the University of New Mexico Human Research Protection Office. A total of 781 pregnant women were enrolled in the NBCS between February 2013 and June 2018. The inclusion criteria for using data from NBCS participants in the current analysis was having developmental screening data from infants between 10 and 13 months and having maternal biospecimen samples collected (*N* = 327 children; 50.2% females). Families whose data were not included in the analysis may not have had biomonitoring data or might have not been able to complete the developmental screening for the time interval that was the focus of the current study.

### 2.2. Procedure and Materials

Participants’ enrollment occurred during pregnancy. Demographic information including maternal age, annual household income, maternal and paternal education, marital status, and maternal employment status was obtained at the time of enrollment and biosamples were collected at the 36-week pregnancy visit or at the time of delivery.

#### 2.2.1. Biospecimen Sample Collection

Urine and blood samples were collected from mothers during the 36-week visit or at the time of delivery using sterile supplies prescreened for metals by the Centers for Disease Control and Prevention (CDC), Division of Laboratory Sciences. These biosamples were processed according to National Center for Environmental Health (NCEH) Methods 3018.3 and 3018A.2 [40] and stored appropriately on site until shipped to the CDC Division of Laboratory Sciences for analysis. Urine and blood samples were processed by the CDC Division of Laboratory Sciences using inductively coupled plasma-dynamic reaction cell-mass spectrometry (ICP-DRC-MS) [41,42,43,44,45,46,47,48]. Biosamples were provided for the following analyses: (1) urinary antimony, total arsenic, and inorganic arsenic compounds including monomethylarsonic acid (MMA) and dimethylarsinic acid (DMA), barium, beryllium, cadmium, cobalt, cesium, iodine, lead, manganese, mercury, molybdenum, platinum, strontium, tin, thallium, tungsten, and uranium; (2) blood lead, cadmium, mercury, selenium, and manganese; and (3) serum copper and zinc. We used blood concentrations of cadmium, lead, and manganese for this analysis because studies have shown that blood measurements are more accurate indicators of exposure to these metals than are urine measurements [49,50].

The following metals were excluded from statistical analysis because they had many observations with values below the limit of detection (LOD): blood and urine mercury (average of methyl and ethyl mercury) with 82% and 51% of samples below LOD, respectively; platinum with 72% of samples below LOD; and beryllium with 83% of samples below LOD. The rest of the metals all had fewer than 20% observations below the LOD. We imputed <LOD values for those metals using the common single-value approach of replacing <LOD with the value of the detection limit divided by the square root of 2 [51]. To account for variations in renal function, urine measurements were normalized to creatinine, expressed as the ratio of metal (in µg) to creatinine (in g), following the imputation of below LOD values [45,52,53,54]. In the sample of (*N* = 327) observations, there were also missing measurements for some metals resulting from random physical loss of sample due to cracking of tubes in transport or damage to sample quality resulting from clotting. This loss accounted for approximately 8% loss of blood samples, 5% of serums, and 14% of urines. Therefore, no systematic missingness was observed for the biomonitoring data.

#### 2.2.2. Neurodevelopmental Assessments

Children’s neurodevelopmental outcomes were assessed during home or clinic visits at approximately the 10- and 13- month assessment windows (+/− 1 month) by trained Navajo research field staff using the Ages and Stages Questionnaire Inventory (ASQ:I) [55]. The ASQ:I is an alternative measure to the commonly used screener Ages and Stages Questionnaire (ASQ) [56,57]. Unlike the binary ASQ-derived score (delayed or not delayed), the ASQ:I yields a continuous score allowing for more quantitative monitoring of developmental progress over time [55]. The ASQ:I has five scales, each with 65–70 items, to assess children’s development in five domains: (1) Communication (e.g., “Does your baby make high-pitched squeals?”); (2) Gross motor (e.g., “When your baby is on her back does she kick her legs?”); (3) Fine motor (e.g., “Does your baby pick up a small toy with only one hand?”); (4) Problem solving (e.g., “Does your baby pick up a toy and put it in her mouth?”); and (5) Personal-social (“Does your baby smile at you”). Items in each domain are hierarchically arranged based on the child’s developmental level necessary to complete the task.

During each home or clinic visit, trained Navajo field staff interviewed the caregiver about the child’s progress in each developmental domain. This procedure was based on our team’s unpublished finding that there were increased levels of item completion and accuracy when staff administered the survey rather than relying solely on parental self-completion. We previously published data on validation of this instrument in a Navajo population relative to the ASQ normative data set [9]. The child was present during survey administration. This allowed the caregiver and field staff an opportunity to directly observe if the child had acquired a specific skill if the caregiver was uncertain about a particular item. Caregivers answered a subset of questions using “2 = yes”, indicating that the child had acquired a skill, “1 = sometimes”, and “0 = not yet”, reflecting that the child had not yet acquired the skill. The subset of questions varied for each participant based on the (1) start point, which was determined by the child’s chronological age, (2) basal point, established by three “yes” responses in a row (if the first three responses were not “yes’, items prior to the start point were administered in reverse order until a basal point was established), and (3) ceiling point, established by achieving three “not yet” responses in a row. Items prior to the established basal point were assumed to be “yes” and items after the ceiling point were assumed to be “not yet” given the hierarchal order of items. The final score for each domain was computed by summing up scores within that domain (item 1 to item corresponding to the established ceiling). Even though the evaluation age windows were only 3 months apart, child’s age is highly predictive of the scores and the prediction curves appear to be nonlinear for communication and personal social. Therefore, for each domain, we performed a nonparametric regression that interpolated a smoothing spline between the final score and child’s ASQ:I assessment age and used the residuals as the age-adjusted scores. These age-adjusted scores were used to represent the current developmental level as the primary outcome measures in all analyses reported here.

### 2.3. Statistical Analysis

First, descriptive statistics for demographics, biomonitoring variables, and ASQ:I scores before age adjustment were generated. Second, we examined associations among biomonitoring variables, between biomonitoring variables and demographics, and between biomonitoring variables and age-adjusted ASQ:I scores. Details about the type of association tests and R implementations used are described in Appendix A.

Third, we identified a set of confounders for each exposure variable (i.e., a metal or a micronutrient measurement) and used the confounders to guide the modeling strategy in the next step. A covariate was defined as either a demographic variable or another biomonitoring variable that was significantly associated (*p*-value < 0.05) with both the exposure and the outcome. These covariates were included as control variables for testing the association between the exposure variables and ASQ:I outcomes, as described below. We proposed a search algorithm for a causal diagram and used the directed acyclic graphs (DAGs) to guide our analyses in obtaining the causal effects of the biomonitoring variables on the outcomes. The search algorithm is presented in Appendix B.

Fourth, we examined the causal effects of each biomonitoring variable on the age-adjusted ASQ:I outcomes assuming a linear relationship between the expected outcome and the independent variables, which included the exposure and its control variables. We used multivariable linear regressions to produce the coefficient estimates for the exposures and the standard errors of the coefficient estimates.

For each ASQ:I domain, we also considered modeling the interactions between the exposure variables as well as the non-linear effects. Since the sample size did not allow for reliable estimation of the interaction effects among all biomonitoring variables simultaneously, we limited the scope of investigation to two-way interactions among biomonitoring variables that had significant causal effects from the multivariable linear regressions in step four and between those biomonitoring variables and metals that are in their control sets. We tested nonlinearity using restricted cubic splines and interactions using expanded models with two-way linear interactions. Generalized additive models were performed to estimate the non-linear and interaction effects. F-tests were performed to evaluate the statistical significance.

Finally, we performed two sensitivity analyses. The first sensitivity analysis set the size of the test in the search algorithm as 0.1 instead of 0.05. The second sensitivity analysis expanded the control set in step four to include all bio-monitoring variables and demographics that significantly associated with either the outcomes or the exposures.

## 3. Results

The distributions of all metals and micronutrients analyzed in the NBCS were compared to the CDC National Health and Nutrition Examination Survey (NHANES) distributions in Table 1. The median and geometric mean levels of cadmium, manganese, uranium, cobalt, barium, copper, antimony, strontium, tin, and tungsten were higher in Navajo pregnant mothers compared to mothers in the NHANES sample. In particular, the median levels of manganese, uranium, cobalt, barium, and tin were more than two times higher in Navajo mothers compared to mothers from the NHANES sample. On the other hand, the median and geometric mean concentrations of lead, arsenic, selenium, thallium, zinc, and iodine were lower in Navajo mothers than mothers in the NHANES sample.

Table 2 presents the demographic characteristics in this study. Of the families in our study, 53.2% had an annual family income less than or equal to $19, 999 (below New Mexico poverty level), 54.7% of the parents had high school or less education, and 67.6% of the mothers were unemployed. NBCS families whose data were included were not significantly different from the families not included in this analysis in terms of demographics listed in Table 1 tests (*p*-values > 0.24 for all comparisons). Informed consent for participation in the study and collection and analysis of biosamples were obtained at the time of enrollment.

Initial correlations between biomonitoring variables and the outcomes are presented in Table 3. Table 4 describes the Pearson’s correlations among bio-monitoring variables. The significance of association tests in Table 3 and Table 4 together were used to inform which metals needed to be controlled for when testing the associations between each individual metal and outcomes. For example, lead, cesium, cobalt, and strontium had significant positive correlations with uranium, suggesting that these metals may share some exposure sources. However, when examining the effects of uranium on a specific outcome, only metals that had significant associations with the outcome of interest were controlled for, i.e., only strontium and arsenic were used as confounding variables when testing the effect of uranium on personal-social skills (see Table A1 in Appendix C).

The comparisons of standardized biomonitoring levels at different demographic subgroups are described in Table 5. The differences were, in general, small with a few exceptions. Mean lead was 0.27 standard deviations higher in mothers with education of high school or below than those with education above high school. The average value of zinc was 0.52 standard deviations lower in women from low-income families (i.e., less than $20,000) than those with higher household income. The average value of selenium was 0.34 standard deviations lower for unemployed mothers compared to employed mothers, and average cesium and thallium were about 0.3 standard deviations higher for women with male babies than those with female babies or women living in households with more than $20,000 annual income compared to those having less household income. The rest of the significant differences were all less than 0.2 standard deviations and, thus, not summarized. Mother’s age had positive associations with cadmium and tungsten levels. Demographic variables were also associated with the age-adjusted ASQ:I scores. Mean age-adjusted fine motor and problem-solving scores were 1.5 points higher for infants from mothers living with a partner. Mean age-adjusted fine motor, personal social, and problem-solving scores were over 1 point higher for infants of mothers who had more than high school education. Mother’s age was also positively associated with children’s problem-solving skills. Although these associations are important and interesting, we did not consider the causal effects of demographics as exposure variables in the current paper.

Using the search algorithm for confounding variables, we identified a set of control variables for each biomonitoring variable for the next step of analyses. The control variables are presented in supplemental Table A1 in Appendix C.

The causal effects of the biomonitoring variables are summarized in Table 6. The sample size for each regression is displayed in parentheses. The values in the column ‘sample std’ represent the sample standard deviations of the biomonitoring values that were used to rescale the variables ((x − mean)/std). For the communication domain, one unit increase in tungsten (about 0.14 μg/g increase in creatinine-adjusted tungsten level) resulted in a 0.76-point increase in the score, whereas one unit increase in molybdenum (about 33.52 μg/g increase in creatinine-adjusted molybdenum level) resulted in a 0.71-point decrease in the score. For the fine motor domain, one unit increase in lead level (1.42 μg/dL) was associated with a 0.90-point decrease in the score, whereas one unit increase in the tungsten level was associated with a 0.57-point increase in the score. For the gross motor domain, there was a negative effect of barium (−0.89) and a positive effect of strontium (0.76), albeit both effects were only marginally significant, with *p*-values < 0.1. For personal-social, copper had a negative linear effect (−1.03) and a sample standard deviation of 45.32 μg/dL. Arsenic and antimony had negative linear effects (with sample standard deviations as 23.99 μg/g and 0.05 μg/g, respectively), and strontium and thallium were found to have positive linear effects on the problem-solving scores (with sample standard deviations of 175.41 μg/g and 0.08 μg/g, respectively).

Some biomonitoring variables that had initial significant associations with ASQ:I outcomes had insignificant causal effects, suggesting that initial associations may be explained by the control variables. For example, cadmium, barium, and arsenic were found to be associated with fine motor, and antimony and tungsten were associated with the problem-solving scores (refer to Table 3). However, these significant associations did not hold in the causal modeling. Further, there were a few biomonitoring variables that did not have significant associations in Table 3 but were significantly associated with the outcome after controlling for confounding factors as this may have increased the power of the hypothesis tests of the coefficients. This included the association of molybdenum with communication and the associations of lead and tungsten with fine motor scores.

Based on models with interactions and splines, we did not find significant evidence for nonlinearity or interaction. Results for tests of non-linearity and interactions are presented in Table A2 in Appendix C. Finally, the results of sensitivity analyses (detailed in Appendix B) were very similar to the main results with one exception where both sensitivity analyses showed significant *p*-values for negative effects of lead on problem-solving scores. The sensitivity analyses relaxed the criteria for including confounding variables in the model to further reduce the potential impact due to residual confounding.

In one sensitivity analysis, the number of confounding variables we controlled for was expanded by changing the *p*-value threshold from 0.05 to 0.1, while the number of control set variables was increased in the second sensitivity analysis by including all bio-monitoring variables and demographics that were significantly associated with either the outcome or the exposure variable (in the original analysis, we only controlled for co-occurring metals that were related to both metals of interest and outcome). As such, when a larger number of confounding variables were controlled for, lead was a significant predictor of problem-solving skills.

## 4. Discussion

The current study was designed to examine the causal effects of maternal prenatal exposure to metals and micronutrients, as measured at the time of delivery, on five infant developmental outcomes between the ages of 10 and 13 months. We developed an algorithm to identify the confounding variables among biomonitoring measurements and demographics for each metal and micronutrient. Our results revealed that prenatal exposures to lead, arsenic, copper, barium, antimony, and molybdenum negatively affected at least one of the ASQ:I domain scores. Surprisingly, tungsten, thallium, and strontium were found to have positive effects on at least one ASQ:I domain scores. Mothers with certain demographic characteristics, particularly those reflective of lower socioeconomic status (e.g., maternal educational attainment, annual income), were at higher risk for metal exposure and for having children with lower ASQ scores. Findings about associations between metal exposures and specific developmental outcomes are detailed below.

### 4.1. Associations between Metal Exposure and Developmental Outcomes

#### 4.1.1. Motor Development

Prenatal exposures to lead and barium were associated with lower motor functioning. The negative effect of lead on young children’s motor functioning, particularly the development of fine motor skills, has been reported in previous research [58,59,60]. It is important to note that this association was observed even though blood lead concentrations in the pregnant mothers in our sample were lower than both those previously reported in NHANES (see Table 1) and CDC recommendations for the health-based limit for blood lead of 5 µg/L. Thus, this finding corroborates the well-documented conclusion that exposure to lead even at levels lower than the U.S. national average and current CDC recommendations can have harmful effects on children’s development and further supports the notion that there are no safe levels of lead in the blood of pregnant woman and their offspring [61].

Evidence for the associations between exposure to barium, a naturally occurring trace element existing mostly in food and drinking water, and children’s neurodevelopmental outcomes is sparse. The potential for human exposure to barium from anthropogenic exposure has increased in the past decades because of the increase in industrial barium uses (e.g., increase in barium levels in water as the result of hydraulic fracturing from shale gas wells) and, hence, more attention has been drawn to examining the potential negative effects of barium exposure on health outcomes [62]. The mean concentration of barium in NBCS mothers was higher than observed in pregnant women in the NHANES sample, suggesting that our population is at risk for barium exposure potentially from mine-waste contamination. Given the association between barium and gross motor skills in the current study, albeit only marginally significant, monitoring how this association unfolds in our sample over time as children develop is warranted.

#### 4.1.2. Cognitive Development

Exposures to arsenic, antimony, and molybdenum were negatively associated with different aspects of infants’ cognitive development. Increased exposure to molybdenum was associated with decreased communication scores and arsenic and antimony exposures were related to decreased problem-solving scores. The negative effects of arsenic exposure at low and moderate levels on children’s cognitive development is well-established [63]. While the concentrations of total arsenic and antimony were lower in our NBCS mothers compared to mothers in the NHANES sample, we previously detected an association between arsenic and elevated oxidative stress, indicating that the observed levels are sufficient for negative biological effects [64]. Total arsenic measurements include organic forms mostly found in foods such as rice, rice-based foods, leafy vegetables, fruit juices, and seafood. Consumption of these foods is not common in the Navajo diet. Indeed, inorganic forms of arsenic and their metabolites (including monomethylarsonic acid) appear to predominate in our sample (potentially through consumption of contaminated water), which are known to be more toxic than organic forms and, hence, may negatively affect neurodevelopmental outcomes [63,64,65,66,67,68]. Thus, this result is consistent with previous research showing that inorganic forms of arsenic are detrimental to neurodevelopmental outcomes [63].

High molybdenum and antimony levels have been reported in children diagnosed with attention problems, such as children diagnosed with attention-deficit /hyperactivity disorder [69,70]. However, these studies either have focused on postnatal exposure to these metals or have been conducted among older, school-aged children. To our knowledge, the current study is the first to address associations between prenatal exposures to these metals and cognitive functioning during infancy. Given that these metals have the potential to transfer from the mother to fetus through the placenta, affecting various aspects of development as early as infancy, more studies need to be conducted to confirm proposed pathways that underlie these associations.

#### 4.1.3. Socioemotional Development

The only metal exposure that negatively predicted infants’ personal-social development in the current study was copper. The main source of copper, an essential element and mineral, is through diet and consumption of foods such as whole grains, nuts, potatoes, and dark leafy greens. Given its nutritional value for the development of fetuses, infants, and young children, most studies have either focused on the positive and nutritional value of copper or on copper deficiency, particularly in combination with deficiency of other micronutrients such as zinc and iron, in relation to developmental outcomes. While serum copper levels are significantly increased during pregnancy and persist throughout pregnancy and early postpartum [71,72,73], a few studies conducted among children and infants have shown that elevated maternal copper concentrations can have negative effects on various aspects of child development [74,75]. A study by Amoros et al. showed a negative linear association between infants’ mental/cognitive scores at 12 months of age and mothers’ serum copper, particularly for those children whose mothers had low iron concentrations [76]. In the current study, we found a linear negative association between copper and infants’ personal and social domain scores during infancy, but no evidence for a non-linear effect to suggest that copper at low levels of concentrations were beneficial for infants’ development. Despite normative changes in copper levels during pregnancy, when compared to NHANES, Navajo mothers had higher concentrations of serum copper and had greater zinc deficiency. High levels of copper combined with decreasing zinc levels in pregnancy have been reported to have negative impacts on the developing fetus [77]. Although we did not find any interaction between copper and zinc in this analysis, the negative effects of copper on infants’ personal-social domain scores may be related to zinc deficiency in the current sample.

#### 4.1.4. Unexpected Findings

Several unexpected findings emerged in the current study that warrant follow-up as we continue data collection on children’s developmental outcomes though middle childhood in this population. Specifically, tungsten was associated with increased communication and fine motor skills, and thallium exposure was associated with an increase in infants’ problem-solving scores. Tungsten and thallium are two heavy metals that have been associated with adverse birth outcomes and negative developmental outcomes, including preterm birth, low birthweight, reduced physical growth during first years of life, and increased risk for attention-deficit/hyperactivity disorders [78,79,80]. Unexpected findings with these two understudied metals may be due to unmeasured differences in confounding variables (e.g., dietary confounders) contributing to the effects of these metals across studied populations. While thallium and tungsten occur naturally in low levels, their presence is increased in water and soils where hard rock mining has occurred and their bioaccumulation in plants is an increasing concern [81,82,83,84]. Therefore, one likely source of these elements is from consumption of home-grown fruits and vegetables, which may introduce other nutritional benefits that we have not captured in the current analysis or previous studies. Future studies should consider exploring confounding dietary variables differing in our population that may explain differences in these associations and use environmental mixture modeling approaches to investigate the impacts of both metal mixtures along with dietary intake on young children’s neurodevelopment.

Another finding that needs to be followed up in future research is the effect of strontium that was positively related to infants’ problem-solving and gross motor skills, even though the geometric mean of strontium was slightly higher in our population than pregnant women in the NHANES sample. The largest source of exposure in the general population to strontium is through drinking water and consumption of food, albeit in small amounts that are not known to be harmful for health [85]. The source of exposure to strontium in our population is not documented and could come from multiple pathways originating from abandoned mine wastes. When strontium enters the body, it mimics and competes with calcium for binding sites and, hence, can accumulate in the bone to negatively affect the bone growth and development [85]. There is sufficient evidence to show that lead also has the potential to replace calcium in the brain, which can result in loss of neurons and decreased communication among neurons through disruptions of calcium effects on stimulation of neurotransmitters. Despite evidence for the negative neural effects of lead replacing calcium in the brain resulting in cognitive delays, developmental studies examining the effects of prenatal exposure to strontium on fetuses’ and young children’s cognitive development have been scarce [86,87,88]. However, our result corroborated data from another recent study showing that low levels of prenatal exposure to strontium were related to increased cognitive scores in a sample of 2-year-old children [89]. Given the relatively high strontium exposure in our population and because of the potential for competing with calcium, more focused investigation of effects of strontium exposure on developmental outcomes in this population is warranted. Future studies also need to consider the role of confounding dietary variables, such as mothers’ multivitamin and calcium intake during pregnancy, as well as potential interactions between exposure to strontium and these confounding factors when examining associations with developmental outcomes.

### 4.2. Associations between Indicators of Socioeconomic Status (SES)

In the current study, mothers’ exposure to metals and micronutrients differed based on their SES. Specifically, mothers with lower education had higher lead exposure, and mothers from low-income families had higher exposures to cesium and thallium. Unemployed mothers and those from low-income families also had lower concentrations of selenium and zinc. Lastly, mothers with lower education had children with lower ASQ:I scores across all domains. Together, these results show that social inequities and vulnerabilities could contribute to increased risk for environmental exposures and negative impacts on developmental outcomes during infancy [90]. A clear understanding of mechanisms and pathways for these findings can help inform the design of interventions as well as public policy for federal and local government and tribal communities.

### 4.3. Limitations, Strengths, and Future Directions

The current study has several limitations. There might be confounders that were not considered in this study, for example, nutrition status and genetic susceptibility, which may affect both the level of the metals and ASQ:I scores. In addition, some studies have shown that exposure to metal mixtures may exacerbate negative neurobiological effects and, hence, more strongly predict deficits in neurodevelopmental outcomes, although the results for specific interaction effects have not always been consistent [63].

Although we evaluated which metals may have confounding effects on metals of interest and controlled for those in our association analyses, a closer look at interactions between metals that co-occur as well as metals and vitamins or other micronutrients that were not considered in the current study should be considered in future research. Evidence suggests that some vitamins or micronutrients (e.g., iron, vitamin C, calcium) can potentiate or neutralize negative effects of toxic metals through their effects on the immune system or other molecular interactions. Further, in the current study, we focused on maternal prenatal exposure as a proxy of exposure, and, thus, future studies should examine the effects of children’s direct exposure using children’s biospecimen data and neurodevelopmental outcomes. Lastly, results from our previous work has shown that women enrolled in the NBCS are representative of pregnant women of childbearing age living across Navajo Nation in terms of median age, family income, and employment status because the majority of births in Navajo Nation occur at the six hospitals participating in the NBCS [10]. Further, our attrition analysis showed that mothers included in our analysis were not different from those not included in terms of basic demographic characteristics. Despite these results, there may be still potential for selection bias because mothers with complete data may have had higher awareness of concerns around environmental exposures across Navajo Nation and their effects on children’s neurodevelopment, resulting in having more intrinsic motivation to respond promptly to data collection demands.

In this study, we examined the direct effects of target metal exposures with appropriate controls for confounding variables using a causal pathway approach. While this type of analytical approach is superior to regression analysis and allows us to examine the unique contribution of each metal to children’s neurodevelopment, examining combinations of metals using metal mixture analytical frameworks is warranted as metals often co-occur and do not occur in isolation. Thus, in our future work, we will examine how interactions between metal mixtures’ exposures and intake of micronutrients and dietary supplements impact neurodevelopment. While generalizability of mixture analysis results is limited by the differences in mixture composition and individual metal concentrations across groups, the causal methods employed in the current analysis can improve our understanding of key components contributing to observed effects, thereby improving our understanding of how metals in mixtures affect health. These analyses controlling for confounders can also help in identifying understudied contributors to observed effects when in the presence of major well-studied metals such as arsenic and lead, helping to overcome the fallacy that a lack of data implies a lack of effect.

Despite these limitations, the current study is well-designed to test for associations between in utero metal exposure and neurodevelopmental outcomes during infancy, as measured by the ASQ:I screening tool. It is focused on young children from non-urban areas in an under-studied, minority population.

## 5. Conclusions

Exposure to metals has been found to adversely affect children’s development. However, most previous studies on metal exposure and neurodevelopmental outcomes focus on older children as the effect of exposure becomes especially strong when it is cumulative or continues over a long period of time [12,22]. By focusing on developmental outcomes during infancy, we found that adverse effects of maternal exposure to metals during pregnancy can start from the early years. Continuing to examine how exposure to metals affects neurodevelopment beyond infancy will be important for understanding the potential risk posed by abandoned mines and waste sites across Navajo Nation on children’s developmental growth and trajectories. The ongoing longitudinal assessment of this sample of children will also help in determining the predictive validity of our early screening assessments and help illuminate the long-term implications of in utero and early childhood exposures.

## Figures and Tables

**Table 1:** Metal and micronutrient concentration distributions in pregnant 14–45-year-old women: NBCS results compared to 2011–2012 and 2013–2014 NHANES data combined.

Metal		LOD (µg/L)	Geometric Mean		50th Percentile		75th Percentile		95th Percentile	
			NBCS	NHANES	NBCS	NHANES	NBCS	NHANES	NBCS	NHANES
**Metals**										
Blood Cadmium (BCD)		0.10	0.31	0.236	0.31	0.25	0.42	0.32	0.72	1.18
Blood Manganese (BMN)		0.99	**24.95**	10.80	25.61	11	30.00	13.4	38.00	22.2
Blood Lead (BPB)		0.07 ^	0.410	0.507	0.37	0.46	0.51	0.63	1.20	2.71
Urine Arsenic (UTAS)		0.26	6.13	7.94	5.88	7.04	8.04	13.40	14.08	55.2
Urine Uranium (UUR)		0.002	**0.02**	0.01	0.02	0.01	0.03	0.01	0.07	0.03
Urine Cobalt (UCO)		0.02	**1.25**	0.69	1.22	0.63	1.77	1.03	2.76	1.88
Urine Barium (UBA)		0.06	**4.35**	1.77	4.19	1.5	10.49	3.66	32.07	11.7
Urine Cesium (UCS)		0.09	4.14	4.77	4.19	4.38	5.26	6.77	7.46	9.83
Serum Copper (SCU)		2.5 ^	245.09	201	250.00	198	280.00	242	330.00	285
Urine Antimony (USB)		0.02	0.07	0.05	0.07	0.05	0.09	0.07	0.18	0.14
Urine Strontium (USR)		2.34	167.47	126	198.23	143	321.87	227	563.45	392
Urine Tin (USN)		0.09	**1.95**	0.54	1.77	0.46	3.57	0.74	12.05	2.19
Urine Thallium (UTL)		0.02	0.14	0.19	0.14	0.19	0.20	0.23	0.30	0.45
Urine Tungsten (UTU)		0.02	0.13	0.08	0.13	0.08	0.20	0.13	0.47	0.24
Urine Molybdenum (UMO)		0.17	45.74	46.10	47.16	44.40	67.53	66.70	107.97	83.8
**Micronutrients**	**Deficiency level**									
Blood Selenium (BSE)	<70 µg/L	24.48	163.45	183	160.00	186	180.00	195	200.00	222
Serum Zinc (SZN)	<66 µg/dL	2.9 ^	52.38	68.3	52.00	68.4	59.00	80	72.50	97.6
Urine Iodine (UIO)	<150 µg/L	2.4	137.283	141.00	126.32	144	229.03	207	561.50	637

Notes. Urine concentrations are µg/g creatinine; blood and serum concentrations are in µg/L, unless specified otherwise. ^ indicates that units are reported in µg/dL. Values indicated in bold indicate metals for which NBCS participants have about two times larger concentrations compared to the National Health and Nutrition Examination Survey (NHANES) sample.

**Table 2:** Demographic characteristics and ASQ:I domain scores for study participants and ASQ:I scores (*N* = 327).

Categorical Variables	*N* (%)
**Annual income**	
Less or equal to $19,999	174 (53.2%)
Above or equal to 20,000	87 (26.6%)
Missing	66 (20.2%)
**Maternal Education**	
High school or below	179 (54.7%)
Above high school	133 (40.7%)
Missing	15 (4.6%)
**Paternal Education**	
High school or below	214 (65.4%)
Above high school	86 (26.3%)
Missing	27 (8.3%)
**Marital status**	
Not married nor living with a partner	57 (17.4%)
Married or living with a partner	262 (80.1%)
Missing	8 (2.4%)
**Mother Employment**	
Employed	100 (30.6%)
Unemployed	221 (67.6%)
Missing	6 (1.8%)
**Continuous Variables**	**Mean (SD); Median [Min, Max]**
**Maternal Age at Birth**	27.4 (5.87); 26.0 (16.0, 42.0)
	**Mean (SD); Min–Max**
ASQ:I Communication	46.00 (6.23); 30–68
ASQ:I Gross Motor	48.00 (7.72); 30–71
ASQ:I Fine Motor	46.60 (5.95); 31–65
ASQ:I Problem-Solving	44.30 (8.58); 12–72
ASQ:I Personal-Social	46.00 (8.40); 26–72

**Table 3:** Pearson’s R correlation between biomonitoring variables and age-adjusted ASQ:I outcomes.

	ASQI COM	ASQI FM	ASQI GM	ASQI PSOC	ASQI PSOL
BCD	0.000	0.070 **	−0.076	−0.032	0.042
BMN	0.043	0.003	0.024	−0.010	0.070
BPB	−0.041	−0.082	0.043	−0.027	−0.040
BSE	0.032	0.048	0.001	0.098 *	0.061
SCU	−0.057	−0.082	−0.022	−0.145 **	−0.068
SZN	0.013	0.055	0.004	−0.004	−0.003 *
UBA	−0.050	−0.132 **	−0.025	−0.066	−0.056
UCO	−0.017	−0.037	0.088	−0.038	0.101
UCS	0.014	0.038 **	0.062	0.030	0.039 *
UIO	0.005	0.036	0.000	−0.020	−0.080
UMO	−0.079	0.065 *	−0.014	−0.053	−0.001 *
USB	−0.065	−0.034	−0.029	−0.069	−0.106 **
USN	0.023	0.072	0.025	0.011	0.073 *
USR	−0.022	0.021 *	0.113 **	−0.030	0.089 **
UTAS	−0.074	−0.156 **	−0.025	−0.100 *	−0.124 **
UTL	−0.009	0.077	0.071	0.008	0.120 **
UTU	0.136 **	0.120 *	0.016	0.047	0.079 **
UUR	−0.053	0.000	0.040	−0.010 *	0.026

Notes. ** *p* < 0.05; * *p* < 0.1. ASQI COM = ASQ:I Communication; ASQI FM = ASQ:I fine motor; ASQI GM = ASQ:I gross motor; ASQI PSOC = ASQ:I Personal-social; ASQI PSOL = ASQ:I problem-solving.

**Table 4:** Correlations and Hoeffding’s Independence Test Table among Metals.

	BCD	BMN	BPB	BSE	SCU	SZN	UBA	UCO	UCS	UIO	UMO	USB	USN	USR	UTAS	UTL	UTU	UUR
BCD	1.000	0.165 **	0.148 **	0.111 **	−0.022	0.036	−0.105	0.240 **	0.088 **	0.023	0.035	−0.055	−0.005	−0.017	−0.049	0.056	−0.052	0.093
BMN	-	1.000	0.145 **	0.195 **	0.214 **	0.157 **	−0.138 **	0.154 **	0.095	−0.088	0.011	−0.170 **	0.050	−0.055	−0.018	0.142 **	−0.065	0.073
BPB	-	-	1.000	0.113 **	−0.026	0.028 **	−0.014	0.548 **	0.677 **	−0.042	0.005	−0.057	−0.058	0.134 **	0.017 **	0.332 **	−0.015	0.339 **
BSE	-	-	-	1.000	0.004	0.219 **	−0.180 **	−0.031	0.169 **	0.062 **	0.000	−0.120	−0.060	−0.030	−0.075	0.167 **	0.010	0.066
SCU	-	-	-	-	1.000	0.118 **	−0.038	−0.037	0.027	−0.061	−0.078	−0.055	0.035	−0.069	0.017	0.046	−0.022	−0.001
SZN	-	-	-	-	-	1.000	−0.127 **	−0.029	0.008	−0.129 **	−0.058	0.027	−0.051	−0.031	−0.070	0.004	−0.052	−0.032
UBA	-	-	-	-	-	-	1.000	0.106 **	0.053	0.166 **	−0.009 **	0.257 **	0.106	0.565 **	0.748 **	0.041	0.001	0.097 *
UCO	-	-	-	-	-	-	-	1.000	0.506 **	0.056	0.234 **	0.080	0.045	0.393 **	0.032 **	0.315 **	0.137 **	0.163 **
UCS	-	-	-	-	-	-	-	-	1.000	0.040 **	0.071 **	0.021	0.068 **	0.254 **	0.056 **	0.566 **	0.041	0.124 **
UIO	-	-	-	-	-	-	-	-	-	1.000	0.100 **	0.187 **	0.069	0.194 **	0.139 **	−0.009 **	0.073 **	0.065
UMO	-	-	-	-	-	-	-	-	-	-	1.000	0.125 **	0.057	0.218 **	0.035 **	0.098	0.311 **	−0.022 **
USB	-	-	-	-	-	-	-	-	-	-	-	1.000	0.095	0.264 **	0.207 **	−0.010	0.055	0.008
USN	-	-	-	-	-	-	-	-	-	-	-	-	1.000	0.030	0.114 **	−0.010	−0.035	−0.038
USR	-	-	-	-	-	-	-	-	-	-	-	-	-	1.000	0.341 **	0.169 **	0.124 **	0.137 **
UTAS	-	-	-	-	-	-	-	-	-	-	-	-	-	-	1.000	0.063 **	0.009 **	0.007 **
UTL	-	-	-	-	-	-	-	-	-	-	-	-	-	-	-	1.000	0.101	0.064
UTU	-	-	-	-	-	-	-	-	-	-	-	-	-	-	-	-	1.000	0.002
UUR	**-**	**-**	**-**	**-**	**-**	**-**	**-**	**-**	**-**	**-**	**-**	**-**	**-**	**-**	**-**	**-**	**-**	1.000

Note. ** *p* < 0.05; * *p* < 0.1;. BCD = Blood Cadmium; BMN = Blood Manganese; BPB = Blood Lead; BSE = Blood Selenium; SCU = Serum Copper; SZN = Serum Zinc; UBA = Urine Barium; UCO = Urine Cobalt; UCS = Urine Cesium; UIO = Urine Iodine; UMO = Urine Molybdenum; USB = Urine Antimony; USN = Urine Tin; USR = Urine strontium; UTAS = Urine Arsenic (total); UTL = Urine Thallium; UTU = Urine Tungsten; UUR = Urine Uranium.

**Table 5:** Relationships of biospecimen and ASQ:I domain scores to demographic variables.

	Male	Female	Not Living with a Partner	Living with a Partner	Maternal Education (<High School)	Maternal Education (>High School)	Below $20,000	$20,000 & Above	Paternal Education (<High School	Paternal Education (>HS)	Not Employed	Employed	Maternal Age
BCD	0.05 *	−0.05	0.03	0.00	0.07	−0.08	0.10	0.06	0.04	−0.04	−0.02	0.05	0.18 **
BMN	0.04	−0.05	−0.06	0.02	0.03	−0.03	−0.08	0.20	0.06	−0.13	−0.02	0.05	0.00
BPB	−0.02	0.03	−0.11	0.03	0.13 **	−0.14	0.02	0.07	0.02 *	0.00	−0.05	0.14	0.05 **
BSE	0.04	−0.04	−0.08	0.03	0.03	−0.03	−0.07	0.09	0.02	0.05	−0.10	0.24 *	0.00
SCU	0.03	−0.03	0.14	−0.04	0.00	−0.03	0.04	−0.01	−0.01	−0.04	0.06	−0.16	0.00
SZN	0.04	−0.05	−0.08	0.03	−0.01	0.04	−0.17	0.35 **	0.04	−0.06	−0.02	0.05	0.05
UBA	0.05	−0.05	0.36	−0.07	0.01	−0.04	0.00	−0.19	0.03	−0.05	0.01	0.00	−0.07
UCO	0.06	−0.05	−0.13	0.02	0.06	−0.08	−0.13	0.20 **	0.01	0.01	−0.03	0.04	0.01
UCS	0.14 **	−0.14	−0.07	0.01	0.00	0.00	−0.11	0.24 **	−0.09	0.25 *	−0.06	0.12	0.08 **
UIO	0.10	−0.09	−0.01	0.00	0.04	−0.07	0.04	−0.07	−0.01	0.04	0.00	0.01	0.08
UMO	−0.02	0.01	−0.03	−0.01	−0.05	0.01	−0.09 *	0.12	−0.09	0.12	−0.06	0.07	0.07
USB	0.01	0.00	−0.10	0.02	0.04	−0.06	0.04 *	−0.05	−0.06	0.17	0.07 **	−0.13	−0.05
USN	0.10	−0.10	0.20 *	−0.03	0.01	−0.06	0.05 **	−0.05	−0.01 *	−0.01	0.03	−0.04	0.00
USR	0.01	−0.01	0.16	−0.03	−0.04	0.05	−0.09	0.12	−0.03	0.11	−0.03	0.07	0.04
UTAS	0.07	−0.06	0.35	−0.07	0.04	−0.05	−0.07	−0.07	0.02	−0.05	0.03	−0.05	−0.05 *
UTL	0.14 **	−0.13	−0.01	0.02	−0.03	0.08	−0.13	0.20 *	−0.06	0.22	−0.04	0.11	0.01
UTU	−0.09 **	0.09	0.00	−0.01	0.04	−0.08	0.08	−0.14	0.05	−0.09	−0.01	−0.01	0.02
UUR	−0.06	0.06	0.14	−0.02	0.05 *	−0.04	−0.01	−0.11	0.02	0.03 **	−0.01	0.04	0.10
ASQI COM	45.77	46.43	45.90	46.17	46.10	46.25	46.10	46.33	46.09	46.29	46.65 **	45.05	−0.04
ASQI FM	46.10	47.11	46.06	46.70	45.95 **	47.49	46.41	47.41	46.45	47.38	46.69	46.32	0.00
ASQI GM	48.31	47.84	49.68	47.66	47.36 *	48.86	47.87	48.71	47.98	48.75	48.02	48.08	−0.02
ASQI PSOC	45.42	46.64	45.98	46.15	45.16 **	47.41	46.08	46.14	45.81	47.06	46.28	45.77	0.02
ASQI PSOL	43.95	44.71	42.88	44.70	43.46 **	45.67	44.05	45.94	44.29	45.34	44.45	44.17	0.05

Notes. ** *p* < 0.05; * *p* < 0.1. ASQI COM = ASQ:I Communication; ASQI FM = ASQ:I fine motor; ASQI GM = ASQ:I gross motor; ASQI PSOC = ASQ:I Personal-social; ASQI PSOL = ASQ:I problem-solving.

**Table 6:** Effects of Metals on Developmental Outcomes.

	Sample Std	COM Estimate	COM Std (*N*)	FM Estimate	COM Std (*N*)	GM Estimate	GM Std (*N*)	PSOC Estimate	PSOC Std (*N*)	PSOL Estimate	PSOL Std (*N*)
BCD	0.23	0.00	0.35 (271)	0.19	0.34 (235)	−0.49	0.38 (293)	−0.31	0.42 (281)	0.36	0.54 (244)
BMN	7.45	0.23	0.32 (271)	−0.06	0.31 (235)	0.16	0.38 (293)	0.08	0.44 (277)	0.41	0.49 (244)
BPB	1.42	−0.21	0.31 (271)	−0.90 **	0.40 (227)	0.20	0.44 (254)	−0.16	0.44 (233)	−0.95	0.63 (234)
BSE	20.00	0.25	0.33 (265)	0.13	0.32 (235)	0.00	0.38 (293)	0.69	0.42 (281)	0.22	0.49 (244)
SCU	45.32	−0.30	0.32 (280)	−0.40	0.29 (284)	−0.14	0.38 (303)	−1.03 **	0.41 (291)	−0.52	0.45 (296)
SZN	11.86	0.07	0.32 (280)	0.22	0.33 (233)	0.03	0.38 (303)	−0.12	0.45 (277)	0.10	0.50 (254)
UBA	17.64	−0.26	0.31 (254)	−0.71	0.55 (235)	−0.89 *	0.48 (276)	0.44	0.69 (242)	−0.35	0.82 (252)
UCO	0.84	−0.17	0.32 (255)	−0.51	0.39 (235)	0.34	0.44 (277)	−0.24	0.44 (265)	0.49	0.56 (267)
UCS	3.40	0.05	0.31 (255)	0.03	0.30 (237)	0.23	0.41 (277)	0.10	0.43 (243)	−0.62	0.55 (267)
UIO	228.76	0.04	0.35 (255)	0.33	0.34 (257)	−0.15	0.41 (277)	0.05	0.50 (243)	−0.48	0.47 (254)
UMO	33.52	−0.71 **	0.34 (255)	0.03	0.34 (257)	−0.27	0.41 (277)	−0.35	0.43 (265)	−0.48	0.50 (254)
USB	0.05	−0.45	0.34 (250)	−0.03	0.33 (257)	−0.42	0.41 (277)	−0.11	0.46 (243)	−0.86 *	0.48 (267)
USN	4.86	0.13	0.36 (255)	0.41	0.33 (250)	0.17	0.42 (277)	0.17	0.45 (265)	0.66	0.51 (260)
USR	175.41	−0.18	0.32 (255)	0.54	0.40 (257)	0.76 *	0.40 (277)	0.05	0.47 (265)	1.26 **	0.51 (267)
UTAS	23.99	−0.38	0.31 (255)	−0.52	0.44 (257)	−0.48	0.42 (277)	−0.68	0.42 (265)	−1.25 **	0.48 (267)
UTL	0.08	−0.11	0.32 (255)	0.35	0.36 (259)	0.35	0.41 (277)	−0.08	0.44 (243)	1.04 *	0.55 (267)
UTU	0.14	0.76 **	0.35 (255)	0.57 *	0.34 (257)	0.02	0.40 (277)	0.37	0.46 (265)	0.57	0.51 (267)
UUR	0.04	−0.26	0.31 (255)	−0.02	0.30 (247)	0.17	0.40 (277)	−0.07	0.42 (255)	−0.04	0.47 (257)

Notes. ** *p* < 0.05; * *p* < 0.1. COM = Communication; FM = Fine Motor; GM = Gross Motor; PSOC = Personal-social; PSOL = Problem-Solving.

**Figure A1:**
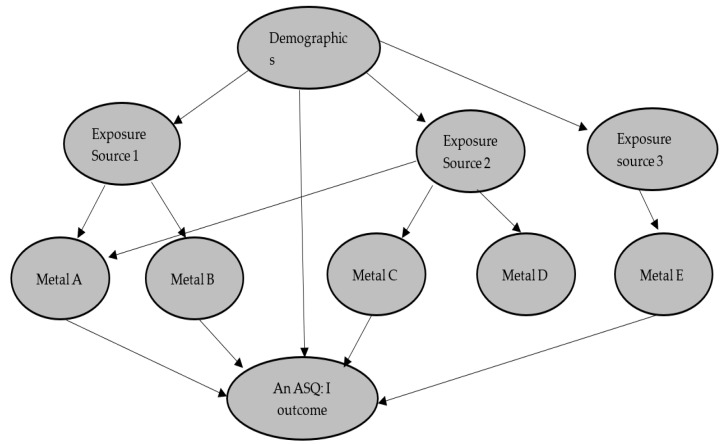
Causal diagram for association between metals and an ASQ:I outcome.

**Figure A2:**
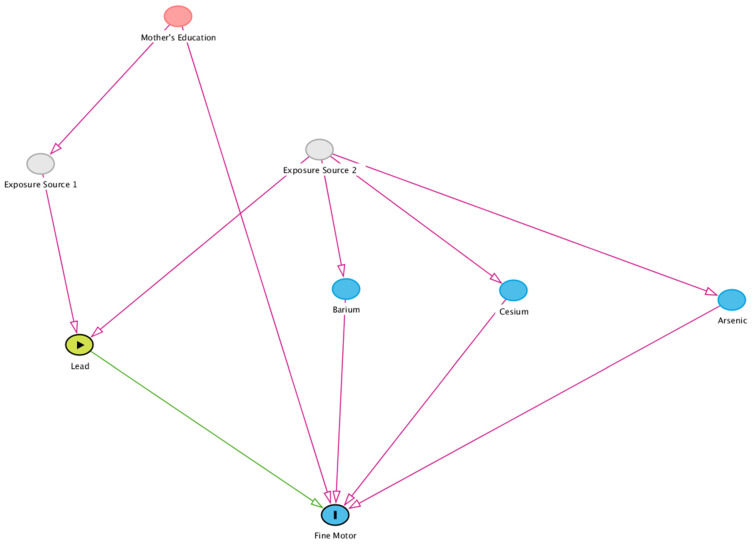
Causal directed acyclic graph for association between lead and fine motor skills.

## Data Availability

Requests for the data presented in this study will require prior approval from the Navajo Nation Human Research Review Board.

## Appendix A. Associations Test

To test significant associations between continuous variables, we performed Monte Carlo permutation tests (R ‘Hmisc’ package, ‘ci.test’ function) on the correlations, where the null hypotheses were that the correlations were zero, and Hoeffding’s independence tests for pairs of continuous variables, where the null hypotheses were that the two variables in the pairs were independent. If either the correlation test or the Hoeffding’s test had a *p*-value less than 0.05, we concluded that there was an association between the two continuous variables. The Monte Carlo permutation test generated the null distribution of the test statistic by permuting the original data and, hence, did not require a specific assumption about the distributions of the continuous variables, and the Hoeffding’s tests were robust to nonlinear associations given that there were some nonlinear associations between some metals and the scores. To test an association between a continuous variable and a categorical variable, we performed a Kruskal–Wallis test (R ‘kruskal.test’ function) where the null hypotheses were that the mean levels of the continuous variable across the different category of the categorical variable were the same.

## Appendix B. Search Algorithm for Confounders

We proposed a search algorithm for a causal diagram that was used to guide for obtaining the causal effects of the biomonitoring variables on the outcomes. A causal diagram describes the causal relationship among variables using directed paths with arrows. We constructed the initial causal diagram based on a priori expert knowledge and used statistical tests of independence to retain or remove variables in the diagram. For example, in Figure A1, the a priori causal diagram included exposure of interest (metal A), a developmental outcome, and exposure sources 1–3, which were confounders for metal A and the outcome. The exposure sources were typically unobservable, but instead we had measurements for co-occurring metals (B, C, D, E) and demographics that can affect the exposures. Figure A2 shows a specific example for the association between lead and fine motor skills. The reason for not being able to identify the exact exposure sources and pathways is that there are more than 500 abandoned mine wastes across Navajo Nation, from which metal compositions can be mobilized via water and air and even enter the food chain. Thus, metal composition and exposure can vary across different sites based on differences in geology, chemistry, and other factors.

An important consideration in obtaining inference of causal relationship based on data collected from observational studies is proper control or adjustment for a set of potential confounder variables. We used a causal DAG to help identify confounders and guide the modeling strategy. The overall strategy is to block all open backdoor paths by controlling for a set of confounding variables in the path [91]. The co-occurring metals and demographic *X* can produce a spurious observed association between *A* and the outcome, i.e., confounding the causal relationship between A and the outcome. To obtain an accurate causal effect of A on the outcome, we can stratify or condition the functional relationship between *A* and the outcome on certain values of these control variables to obtain a relatively accurate and unbiased estimate. According to the backdoor criterion [91,92,93], the control variables for *A* need to block the backdoor paths, which enter both *A* and the outcome while connecting the two variables. In the example described in Figure A1, since the source exposures were unobserved, the control variable sets for *A* could be {B, C, X} or {B, C, X, E}. We proposed the following algorithm to identify the control variable set for metal *A:* We included all biomonitoring variables and demographics that associated significantly with both the outcome and *A.* If the *p*-value of an association test was less than 0.05, we concluded there was a significant association. Using this algorithm, we may identify (B,C,X,D,E) as the control variables for *A* because *D* was also a confounder that can be significantly associated with both the outcome and *A*. The association between *D* and the outcome was merely induced by the exposure source 2, which affected both the outcome and *D*. It was possible to identify a sufficient set with fewer numbers of control variables for estimation of the causal effects. Additional testing for conditional independence between *D* and the outcome (conditioning on *X* and *C*) and between *A* and *E* (conditioning on *X*) may remove *D* and *E* from the control set. However, an exhaustive search among all relevant conditional independence tests would be needed to identify *D* and *E*. Moreover, including more variables that are not mediators between *A* and the outcome does not create bias for the causal inference. Therefore, we did not perform conditional independence tests to remove *D* and E in our algorithm, and instead adjusted for the larger set of confounder variables to obtain causal inference.

Since type II errors can occur for hypothesis tests, we may identify fewer control variables than {B, C, X}. We considered two sensitivity analyses. One sensitivity analysis changed the threshold for *p*-value from 0.05 to 0.1. The second sensitivity analysis expanded the control set to include all bio-monitoring variables and demographics that were significantly associated with either the outcome or the exposure variable, according to the disjunctive cause criterion by VanderWeele and Shpitser [93]. The basic idea of the disjunctive cause criterion is controlling for each covariate that is a cause of the exposure, or of the outcome, or of both, excluding from this set any variable known to be an instrumental variable and including as a covariate any proxy for an unmeasured variable that is a common cause of both the exposure and the outcome.

## Appendix C. Appendix Table

**Table A1:** The sets of control variables based on a threshold of 0.05 for the *p*-values.

	ASQI Com	ASQI FM	ASQI GM	ASQI PSOC	ASQI PSOL
**BCD**		UCS			
**BMN**		BCD, UBA		SCU	USB
**BPB**		BCD, UCS, UTAS, Mother’s education	USR		USR, UTAS
**BSE**		BCD, UBA, UCS			
**SCU**					
**SZN**		UBA		SCU	
**UBA**		UTAS	USR		USB, USR, UTAS
**UCO**	UTU	BCD, UBA, UCS, UTAS	USR		USR, UTAS, UTU
**UCS**		BCD, UTAS	USR		USR, UTAS
**UIO**	UTU	UBA, UCS, UTAS	USR		USB, USR, UTAS, UTU
**UMO**	UTU	UBA, UCS, UTAS	USR		USB, USR, UTAS, UTU
**USB**		UBA, UTAS	USR		USR, UTAS
**USN**		UCS, UTAS			UTAS
**USR**	UTU	UBA, UCS, UTAS			USB, UTAS, UTU
**UTAS**	UTU	UBA, UCS	USR		USB, USR, UTU
**UTL**		UCS, UTAS	USR		USR, UTAS
**UTU**		UTAS	USR		USR, UTAS
**UUR**		UCS, UTAS	USR		USR, UTAS

**Table A2:** List of *p*-values with multiple comparison adjustments for testing the non-linear and interaction effects of metals on ASQ.

	*p*-Value	Adjusted *p*-Value
**Communication**		
UTU (nonlinear)	0.35	0.35
UMO (nonlinear)	0.04	0.09
UTU × UMO	0.93	0.93
**Fine Motor**		
BPB (nonlinear)	0.39	1.00
UTU (nonlinear)	0.15	0.89
BCD (nonlinear)	0.55	1.00
UCS (nonlinear)	0.18	0.89
UBA (nonlinear)	0.99	1.00
UTAS (nonlinear)	0.32	1.00
BPB × BCD	0.90	1.00
BPB × UCS	0.50	1.00
BPB × UTAS	0.75	1.00
UTAS × UTU	1.00	0.59
BCD × UCS	0.05	0.36
UCS × UTAS	0.66	1.00
UBA × UTAS	0.36	1.00
**Gross motor**		
USR (nonlinear)	0.86	0.86
UBA (nonlinear)	0.26	0.52
USR × UBA	0.51	0.51
**Problem-solving**		
USR	0.58	1.00
UTAS	0.91	1.00
USB	0.31	0.94
UTU	0.08	0.31
USR × UTAS	0.66	1.00
USR × USB	0.84	1.00
USR × UTU	0.612	1.00
UTAS × USB	0.436	1.00
UTAS × UTU	0.510	1.00

Notes. Adjusted *p*-values are Family-Wise Error Rate *p*-values adjusted for multiple comparisons to control for Type I error using the Holms’ method; multiple testing were done separately for non-linearity and interaction tests. No test was conducted for personal-social because SCU was the only metal with a significant main effect.
